# Supplementary material for: Sex and region-specific disruption of autophagy and mitophagy in Alzheimer’s disease: linking cellular dysfunction to cognitive decline
Source: Cell Death Discov. 2025 Apr 26;11:204. doi: 10.1038/s41420-025-02490-0 (PMC12033262; doi:10.1038/s41420-025-02490-0)
Supplement: Supplementary file 1 — Supplemental Figures Legends and Justification of Sample Size [file 41420_2025_2490_MOESM1_ESM.docx]

**Figure S1. Association of Autophagosome with Memory Impairment** **(A, B)** Association of LC3B-II levels (autophagosome) with the NOR and NOP test in females, respectively. In females, no association was found between autophagosome and memory deficit (*P* ˃ 0.05).

**Figure S2. Association of Autophagosome with Memory Impairment** **(A, B)** Autophagosome degradation (SQSTM1) level association with the NOP test in females and males. Our analyses reveal no significant association between autophagosome degradation levels and the NOP test in 3xTg-AD mice (*P* ˃ 0.05). **(C, D)** SQSTM1 level association with the NOR test in females and males. Our analyses reveal no significant association between SQSTM1 levels and NOR test in 3xTg-AD mice (*P* ˃ 0.05). Statistical analyses were conducted using Spearman's correlation test, with a *P*-value < 0.05 considered statistically significant. LC3B-II, microtubule-associated protein 1 light chain 3 beta-II; SQSTM1, sequestosome 1; NOR, novel object recognition; NOP, novel object placement.

**Figure S3. Association of BNIP3L monomer Expression Involved in Mitophagy with Memory Impairment** **(A, B)** Correlation of NOR test with the levels of BNIP3L monomer in the cortex and hippocampus of females and males. **(C, D)** Correlation of NOP test with the levels of BNIP3L monomer in the cortex and hippocampus of females and males. **No association was found between the monomer forms of** BNIP3L **and memory impairment (*P* ˃ 0.05).**

**Figure S4. Association of Mitochondria and Mitophagosome Numbers with NOR Memory Impairment**. **(A, B)** Correlation of NOR test with mitophagosome and mitochondria number in the cortex and hippocampus of male 3xTg-AD mice. Results show no correlation between mitophagosome and mitochondria number and cognition memory in male mice (*P* ˃ 0.05). Statistical analyses were conducted using Spearman's correlation test to determine statistical significance, with a *P*-value < 0.05 considered statistically significant. BNIP3L, BCL2/adenovirus E1B interacting protein 3-like; BNIP3, BCL2/adenovirus E1B NOR, novel object recognition; NOP, novel object placement.

**Figure S5. Association of Mitochondria and Mitophagosome Numbers with NOP Memory Impairment**. **(A, B)** Correlation of NOP test with mitophagosome number in the cortex and hippocampus of female and male 3xTg-AD mice. **(C, D)** Correlation of NOP test with mitochondria number in the cortex and hippocampus of female and male 3xTg-AD mice. Our investigation showed no significant association of mitochondria and mitophagosome number with spatial memory (*P* ˃ 0.05). Statistical significance was tested using Spearman’s correlation test. A *P*-value < 0.05 was considered statistically significant. NOR, novel object recognition; NOP, novel object placement.

**Figure S6. Association of BNIP3L dimer Protein Expression Involved in Mitophagy with Memory Impairment** **(A, B)** Correlation of BNIP3L dimer with NOR test in the cortex and hippocampus of females and males. **No association was found between the dimer forms of** BNIP3L **and cognition memory deficit (*P* ˃ 0.05).**  **(C)** Correlation of BNIP3L dimer with NOP test in the cortex and hippocampus of males. Analysis shows no association of spatial memory with BNIP3L expression (*P* ˃ 0.05). Statistical analyses were conducted using Spearman's correlation test to determine statistical significance, with a *P*-value < 0.05 considered statistically significant. BNIP3L, BCL2/adenovirus E1B interacting protein 3-like; NOR, novel object recognition; NOP, novel object placement.

**Figure S7. Association of BNIP3 monomer Protein Expression Involved in Mitophagy with Memory Impairment** **(A, B)** Correlation of NOR test with the expression levels of BNIP3 monomer in the cortex and hippocampus of female and male 3xTg-AD mice. **(C,D).** Correlation of NOP tests with the expression levels of BNIP3 monomer in the cortex and hippocampus of female and male 3xTg-AD mice. Our investigation shows no significant correlation between monomer of BNIP3 monomer expression and memory deficit (*P* ˃ 0.05). Statistical analyses were conducted using Spearman's correlation test to determine statistical significance, with a *P*-value < 0.05 considered statistically significant. BNIP3, BCL2/adenovirus E1B interacting protein 3; BCL2L13, BCL2 like 13; NOR, novel object recognition; NOP, novel object placement.

**Figure S8. Association of BNIP3 dimer Protein Expression with Memory Impairment** **(A-B)** **(C, D)** Correlation of NOR and NOP test with the expression levels of BNIP3 dimer in the cortex and hippocampus of female and male 3xTg-AD mice. Our investigation shows no significant correlation between dimer of BNIP3 expression (mitophagy) and memory deficit (*P* ˃ 0.05). Statistical analyses were conducted using Spearman's correlation test to determine statistical significance, with a *P*-value < 0.05 considered statistically significant. BNIP3, BCL2/adenovirus E1B interacting protein 3; BCL2L13, BCL2 like 13; NOR, novel object recognition; NOP, novel object placement.

**Figure S9. Association of BCL2L13 Protein Expression with Memory Impairment** **(A, B)** Correlation of BCL2L13 levels with NOP test in females and males. Our analysis shows no association between BCL2L13 levels and memory deficit (*P* ˃ 0.05). **(C)** Correlation of BCL2L13 levels with NOR test in male. No correlation was found between BCL2L13 and cognition memory in males (*P* ˃ 0.05). Statistical analyses were conducted using Spearman's correlation test to determine statistical significance, with a *P*-value < 0.05 considered statistically significant. BNIP3L, BCL2/adenovirus E1B interacting protein 3-like; BNIP3, BCL2/adenovirus E1B interacting protein 3; BCL2L13, BCL2 like 13; NOR, novel object recognition; NOP, novel object placement.

**Justification of Sample Size**:

The sample sizes for this study were chosen based on balancing scientific rigor with ethical considerations, as well as feasibility within the experimental framework.

1. **Sample Size Justification for WB and TEM (n=3-4)**:
   - **Western Blot (WB) and Transmission Electron Microscopy (TEM)** are commonly performed with lower sample sizes (typically n=3-4) in exploratory studies of this nature. Our choice of n=3-4 aligns with established practices in these techniques, where consistency and reproducibility in visual representation and quantitative data across multiple runs serve as reliable indicators of effect size and biological significance.
   - Given the constraints of WB and TEM assays, which are sensitive to both technical variability and tissue limitations, larger sample sizes may introduce greater technical and resource challenges without proportionate increases in detection power for these assays. Therefore, n=3-4 was chosen as it provides sufficient power to detect large and biologically relevant changes while ensuring ethical resource use.
2. **Sample Size Justification for Behavioral Studies (n=5)**:
   - In behavioral studies, the choice of **n=5** reflects a balance between ethical animal use and adequate statistical power to detect meaningful behavioral differences. The sample size was informed by pilot studies and established literature in behavioral science, which frequently use n=4-6 in rodent models to reliably detect significant behavioral alterations with moderate to large effect sizes.
   - Although no formal power calculation was conducted for this exploratory phase, the use of n=5 is supported by previous research indicating that this sample size is sufficient to capture expected effect sizes in similar behavioral contexts while minimizing potential distress and resource use.
3. **Ethical and Feasibility Constraints**:
   - Recognizing the ethical considerations in animal research, particularly in accordance with the **3Rs principle** (Reduction, Refinement, Replacement), sample sizes were determined to minimize the number of animals used while ensuring robust and reproducible results. Larger sample sizes would require excessive animal use without significantly improving data quality or statistical power in this initial exploratory phase.

For more comprehensive studies or confirmatory phases, we anticipate adjusting sample sizes in accordance with formal power calculations to validate and expand upon these preliminary findings. We appreciate any additional guidance to further refine our approach to sample size estimation.

**Randomization**:

In this study, we used a non-randomized assignment approach for the 3xTg and C57BL/6 mice due to the specific scientific objectives related to genotype-based comparison and sex-specific analyses. The decision to forgo randomization was intentional to maintain control over genotype and sex variables, which are critical to understanding the effects of the APPSwe, PSEN1M146V, and MAPT/TauP301L mutations on male and female phenotypes. By directly comparing defined groups (3xTg vs. C57BL/6; male vs. female), we ensured that each experimental condition had a balanced and controlled representation, thereby reducing confounding factors associated with variable group compositions.

Moreover, the study utilized genetically identical animals maintained under uniform environmental conditions, minimizing potential bias that randomization typically addresses. This controlled, consistent assignment allows for rigorous genotype and sex-specific comparisons and aligns with similar established practices in genetic model studies. Future confirmatory studies may include randomization when assessing broader or less controlled variables, but it was deemed unnecessary for this phase, which required strict control of genotype and sex factors.

This approach, while non-randomized, follows established standards for targeted genetic model research and has been reviewed and approved by the University of Manitoba Animal Care and Use Committee, ensuring compliance with the Canadian Council on Animal Care and ARRIVE guidelines.

**Blinding Protocol**:

In this study, blinding procedures were rigorously implemented across multiple stages to minimize potential bias and ensure objective data assessment. Specifically:

1. **Transmission Electron Microscopy (TEM) Analysis**:
   - For TEM analysis, samples were assigned blind to the expert responsible for quantification. This process involved labeling each sample with a unique code, which concealed the group allocation (genotype and sex) from the individual performing the analysis. By ensuring that the expert was unaware of the group identities, we minimized any subjective bias in the quantification process, allowing for unbiased assessment of ultrastructural features.
2. **Correlation Analysis and Machine Learning**:
   - For both correlation analyses and machine learning applications, sample data were provided to the respective analysts through coded identifiers, maintaining blinding to group allocation. Each sample was assigned an anonymized code by a third party before being submitted for analysis, ensuring that neither genotype nor experimental conditions influenced the analyst's interpretation or model training. This blinding allowed for objective computational analysis and model training, crucial for the integrity of these data-driven assessments.
3. **Behavioral and Western Blot (WB) Analyses**:
   - Blinding was also applied to behavioral assessments and Western Blot analyses. In the case of behavioral studies, data acquisition and scoring were performed by personnel who were blinded to the animals’ genotype and treatment groups. This ensured that behavioral observations were unbiased and solely reflective of the animals’ actions rather than any preconceived expectations from the researchers. Similarly, for WB analysis, samples were processed and labeled with unique codes that masked group identities before protein quantification, thereby maintaining blinding throughout data collection and quantification.

These blinding protocols were implemented across all critical stages of analysis to maintain scientific rigor and integrity in data assessment. The blinding methods are described in [specific section/paragraph/page of the supplementary materials], where further details of the coding and assignment process are outlined.

**Statistical Analysis Justification and Assumptions**

1. **Justification of Statistical Tests**
   In our study, statistical analyses were carefully selected to align with the nature of our data and the experimental design. **Two-way ANOVA** was applied to compare differences in protein expression levels (Western blot), autophagy and mitophagy markers (TEM quantification), and cognitive outcomes (behavioral tests) across genotype and sex groups. This test is appropriate for detecting main effects and interactions between independent variables (genotype and sex) on dependent variables. For correlation analyses, **Spearman’s correlation test** was employed, which is suitable for assessing relationships within non-normally distributed data, making it ideal for our exploratory analyses of protein expression and behavioral performance.
2. **Assumptions of Normality**
   Although no formal normality tests (e.g., Shapiro-Wilk test) were conducted, the use of two-way ANOVA assumes that the data are approximately normally distributed. In biological studies like ours, data such as protein quantification and behavioral scores typically follow a near-normal distribution, especially when sample sizes are balanced across groups (n=5 per sex and genotype group). This assumption is supported by our data distribution across multiple replicates, making ANOVA a suitable test for our comparisons.
3. **Estimates of Variation Within Each Group**
   We have included **standard deviation (SD)** as an estimate of variation within each group across all quantifications, including Western blot results, TEM measurements, and behavioral test scores. SD values are presented as error bars in the figures, providing a clear indication of the data spread within each group. This helps illustrate the consistency and reliability of the measurements for each experimental condition, supporting the validity of our statistical comparisons.
4. **Homogeneity of Variance Across Groups**
   Although no formal test for homogeneity of variance (e.g., Levene's test) was performed, we examined the SD visually across groups in each figure. The error bars indicate comparable variance levels among groups, suggesting that the assumption of homogeneity holds reasonably well. The consistency of experimental conditions and sample sizes (n=5 for each sex and genotype) further supports the expectation of similar variance across groups. Therefore, the variance differences, if present, would likely have minimal impact on the robustness of the ANOVA results.
