# Supplementary figures and images for: Sex and region-specific disruption of autophagy and mitophagy in Alzheimer’s disease: linking cellular dysfunction to cognitive decline
Source: Cell Death Discov. 2025 Apr 26;11:204. doi: 10.1038/s41420-025-02490-0 (PMC12033262; doi:10.1038/s41420-025-02490-0)

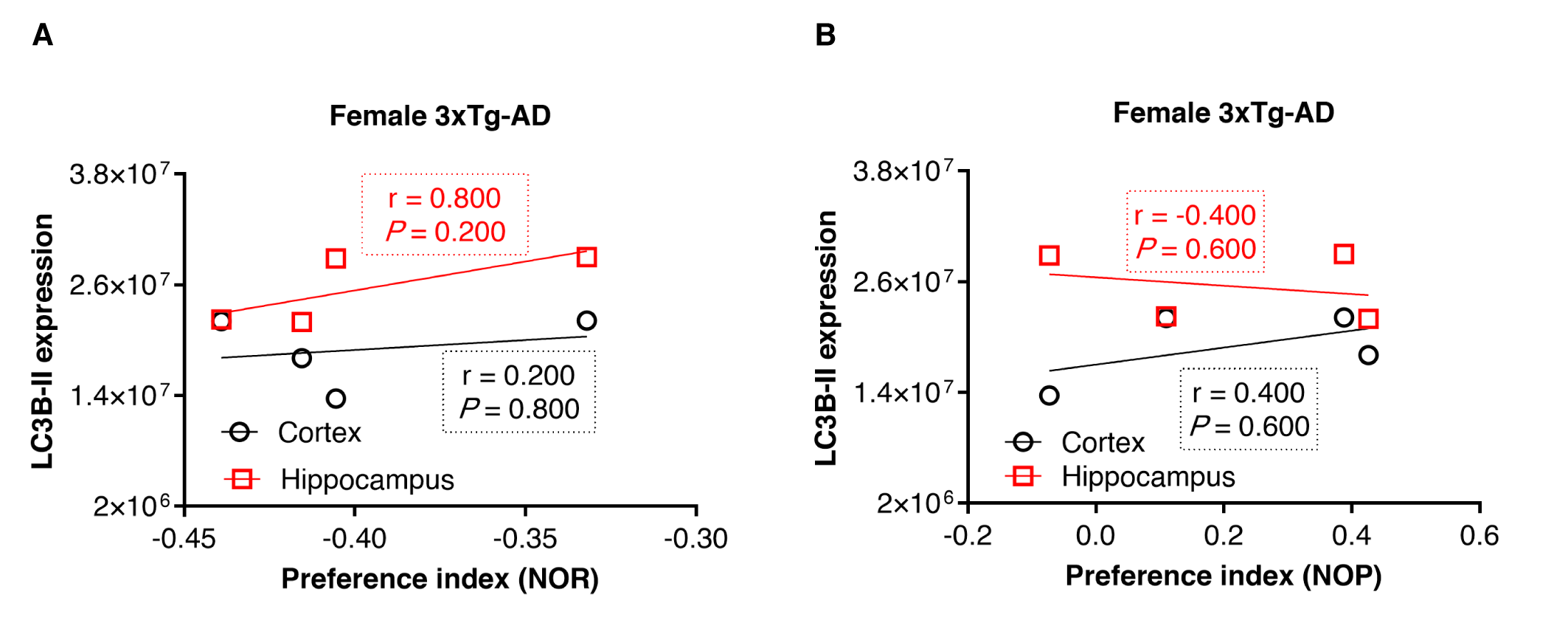

Supplement: Supplementary file 2 — Supplementary Figure 1 [file 41420_2025_2490_MOESM2_ESM.tif]

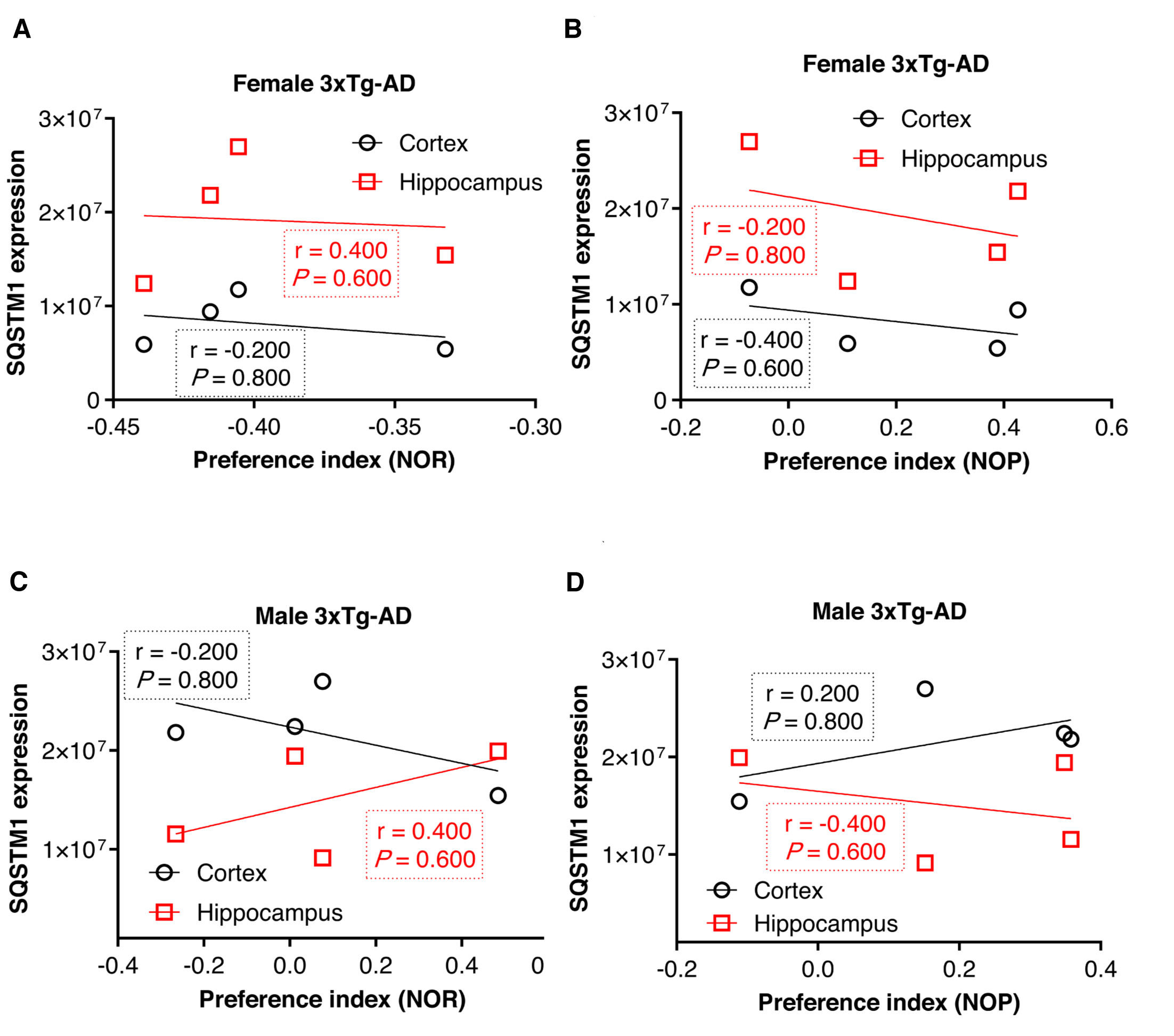

Supplement: Supplementary file 3 — Supplementary Figure 2 [file 41420_2025_2490_MOESM3_ESM.tif]

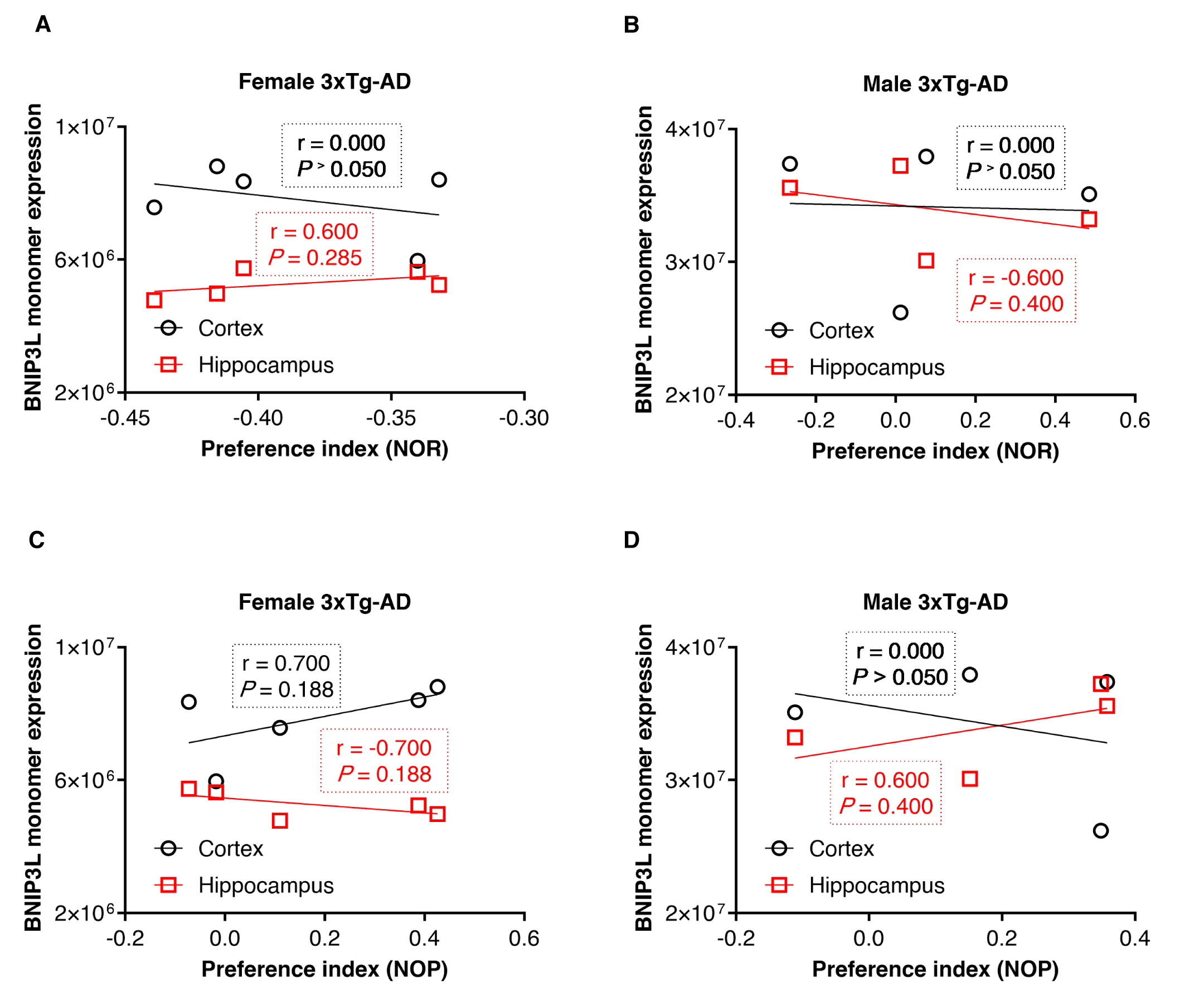

Supplement: Supplementary file 4 — Supplementary Figure 3 [file 41420_2025_2490_MOESM4_ESM.tif]

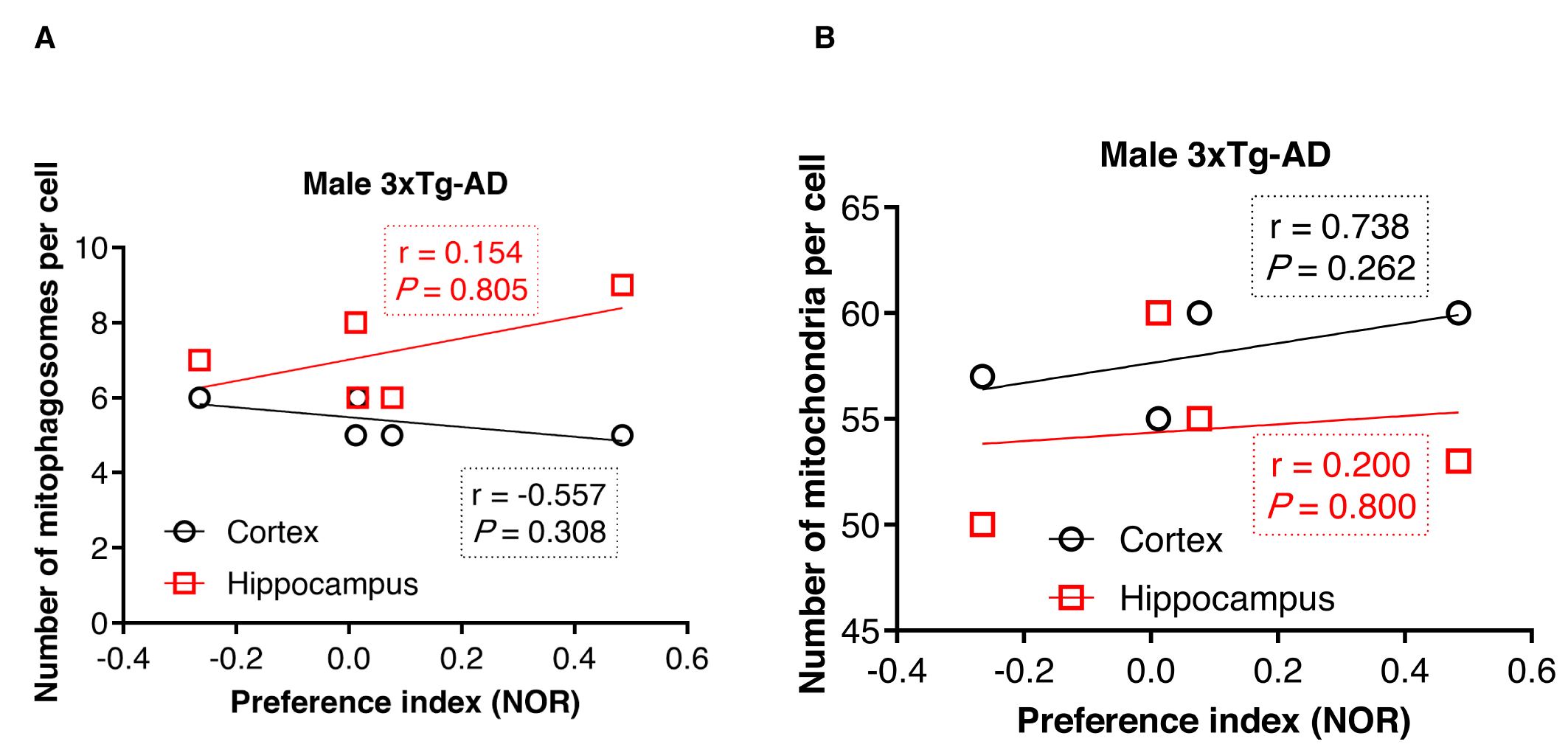

Supplement: Supplementary file 5 — Supplementary Figure 4 [file 41420_2025_2490_MOESM5_ESM.tif]

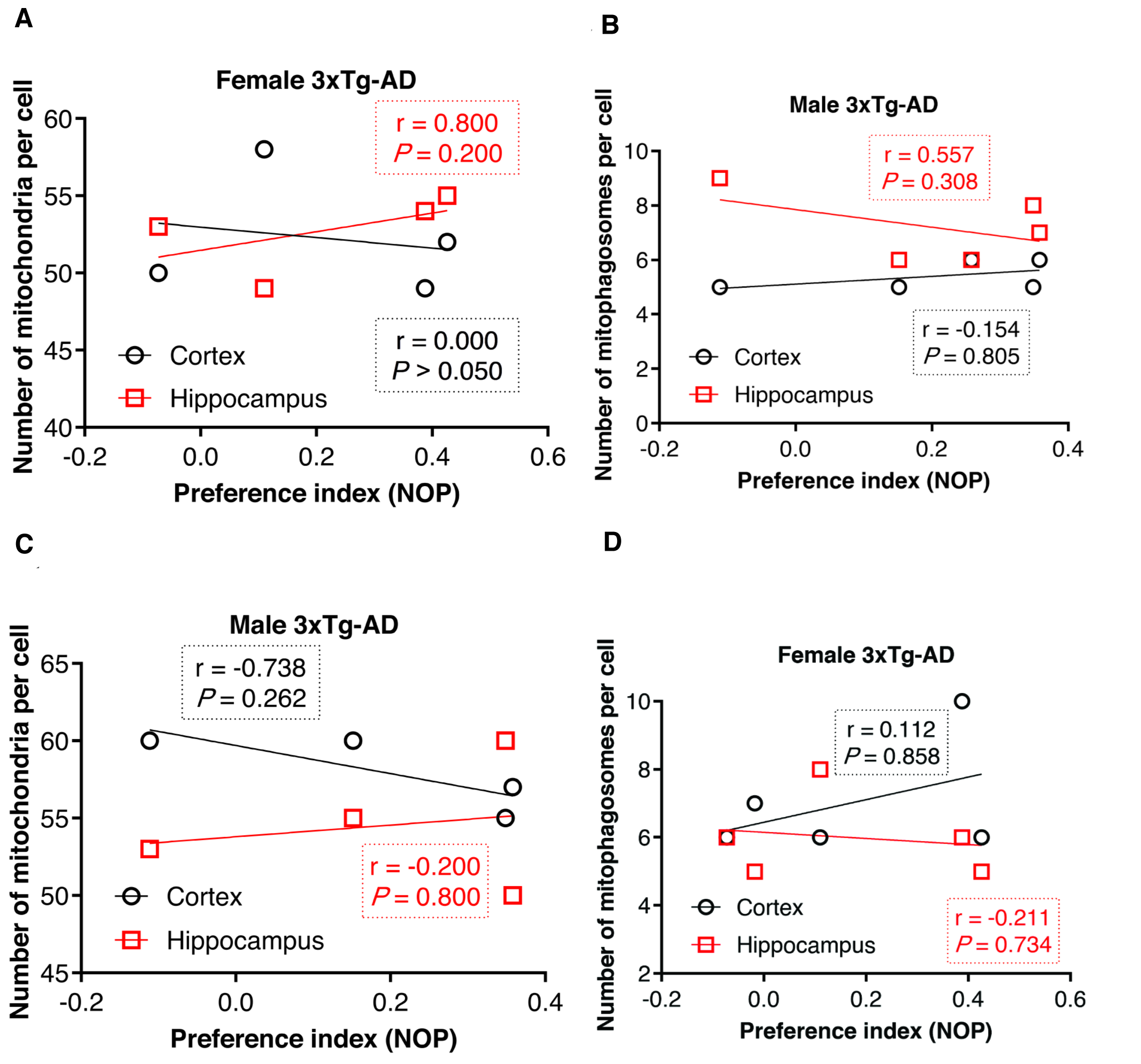

Supplement: Supplementary file 6 — Supplementary Figure 5 [file 41420_2025_2490_MOESM6_ESM.tif]

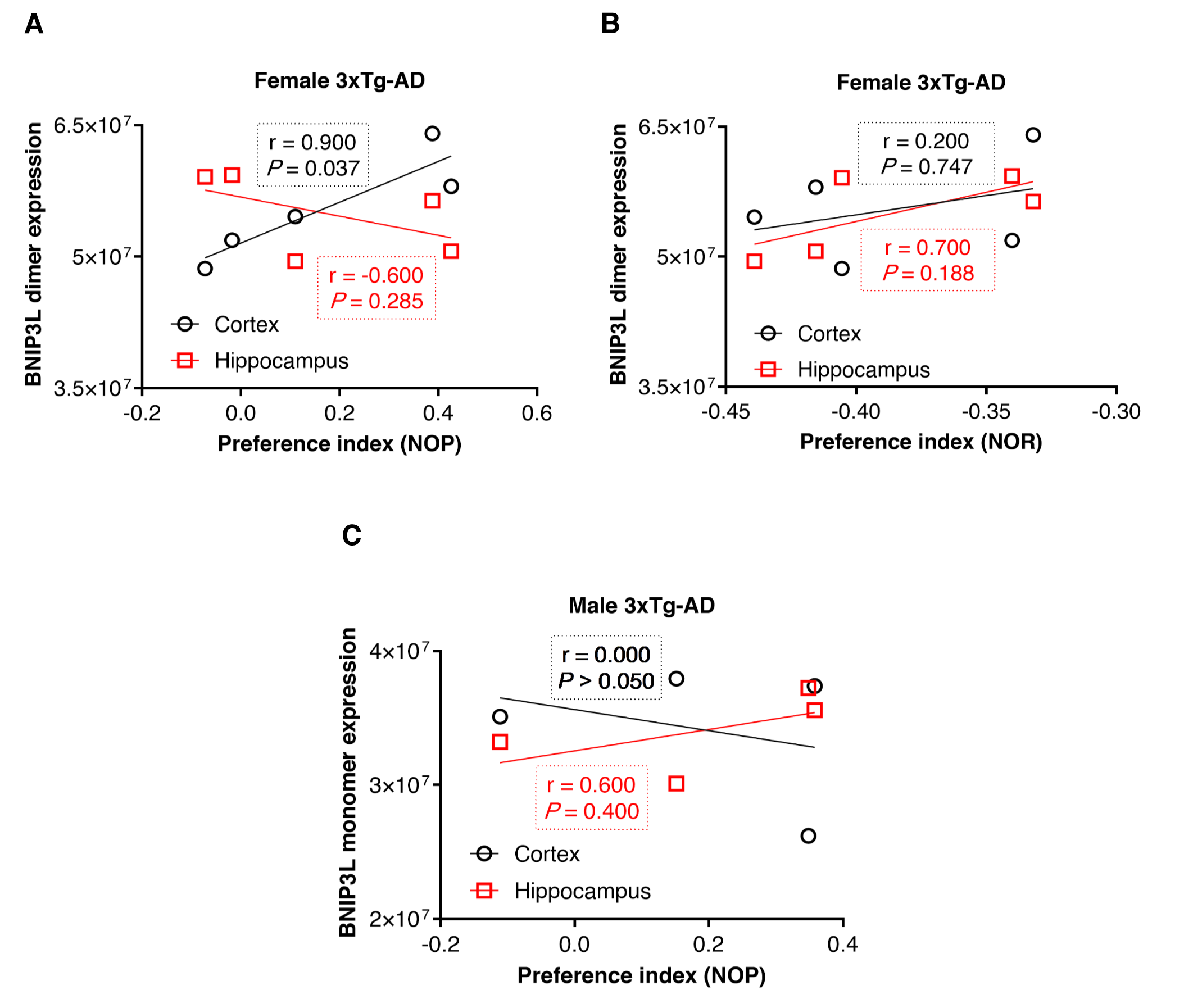

Supplement: Supplementary file 7 — Supplementary Figure 6 [file 41420_2025_2490_MOESM7_ESM.tif]

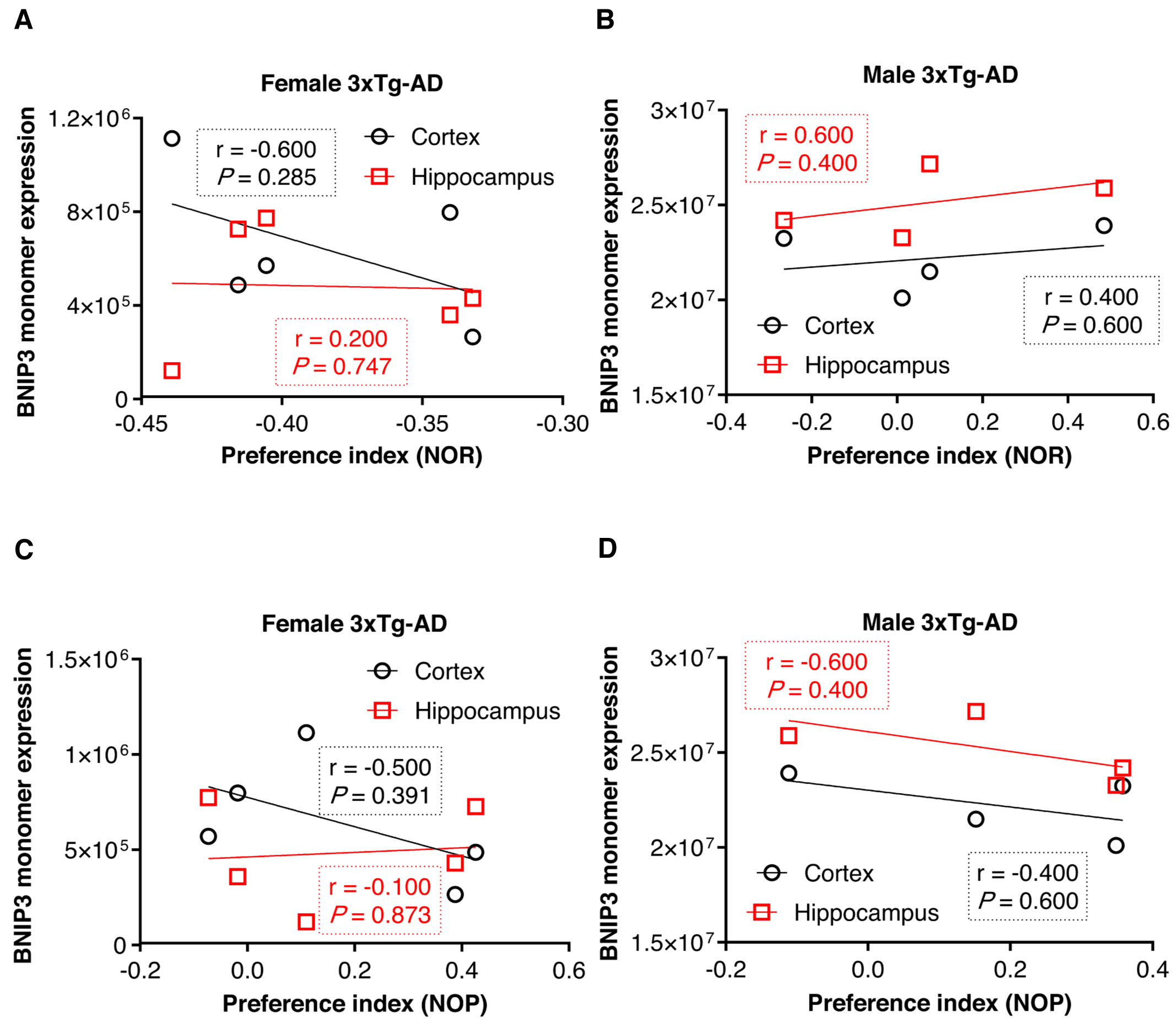

Supplement: Supplementary file 8 — Supplementary Figure 7 [file 41420_2025_2490_MOESM8_ESM.tif]

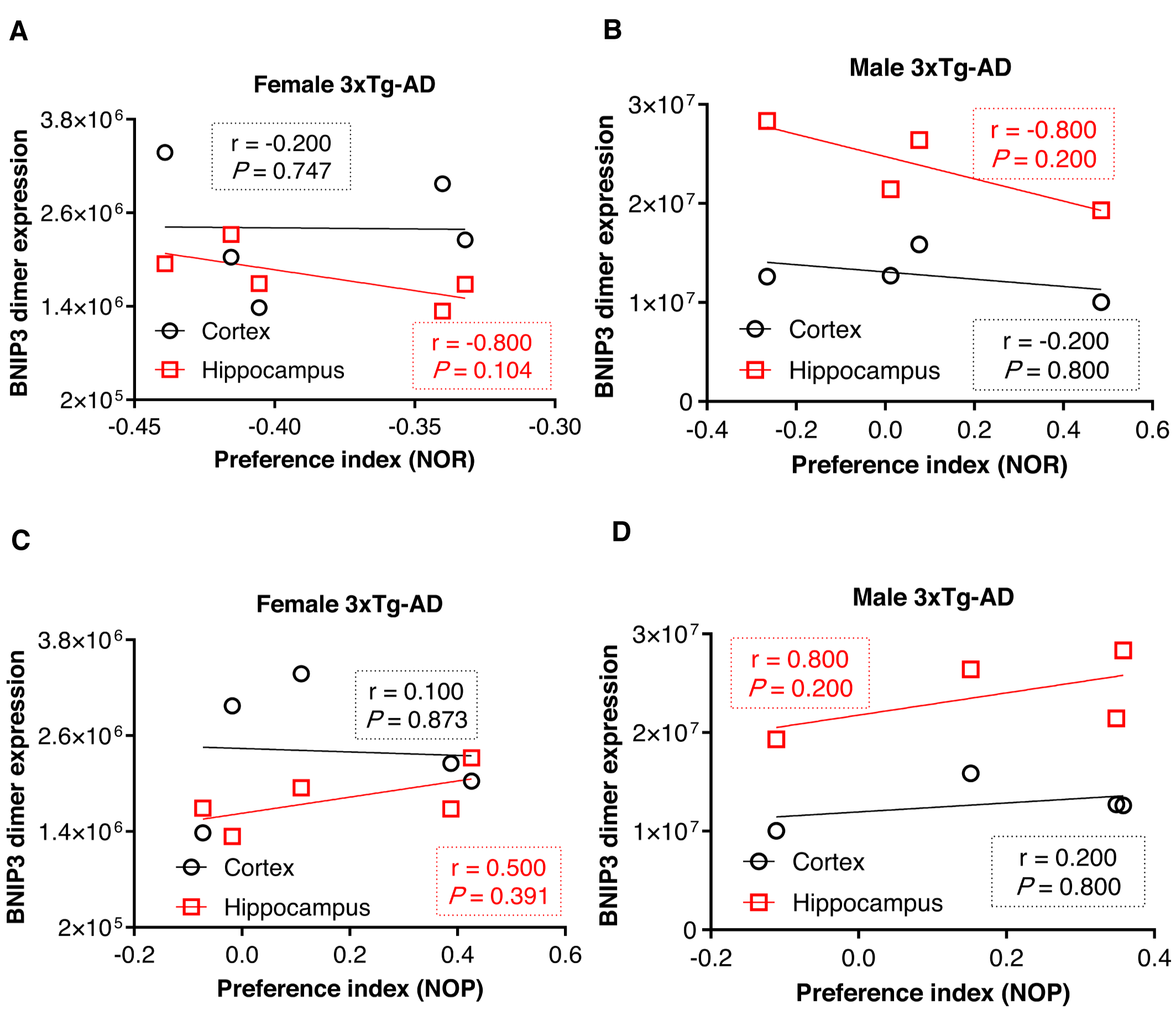

Supplement: Supplementary file 9 — Supplementary Figure 8 [file 41420_2025_2490_MOESM9_ESM.tif]

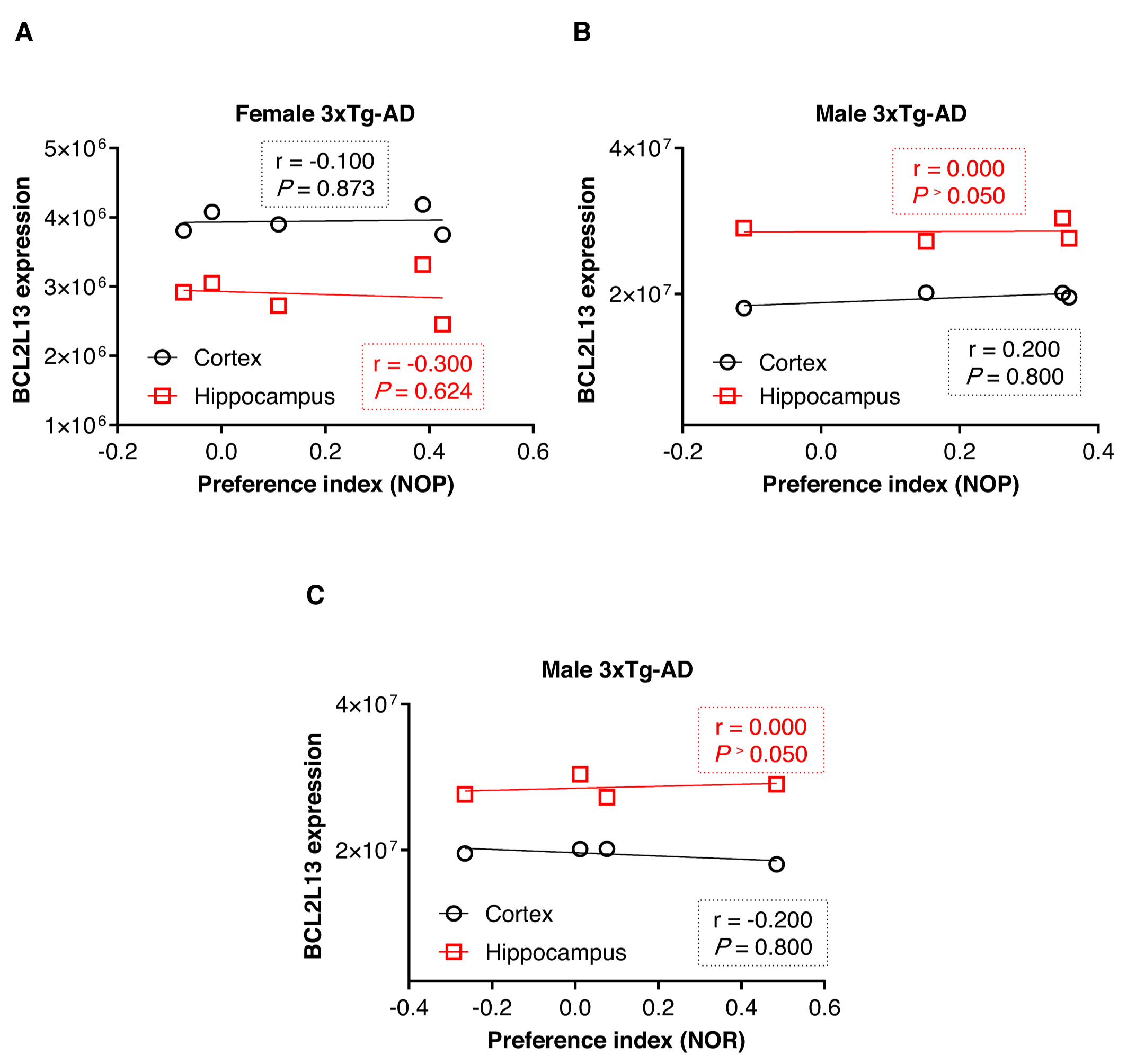

Supplement: Supplementary file 10 — Supplementary Figure 9 [file 41420_2025_2490_MOESM10_ESM.tif]
