# Supplementary material for: Sex and region-specific disruption of autophagy and mitophagy in Alzheimer’s disease: linking cellular dysfunction to cognitive decline
Source: Cell Death Discov. 2025 Apr 26;11:204. doi: 10.1038/s41420-025-02490-0 (PMC12033262; doi:10.1038/s41420-025-02490-0)

BCL2L13 FEAMLE TOTAL PROTEIN

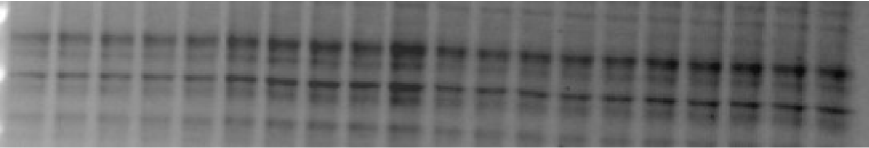

BCL2L13 FEMALE

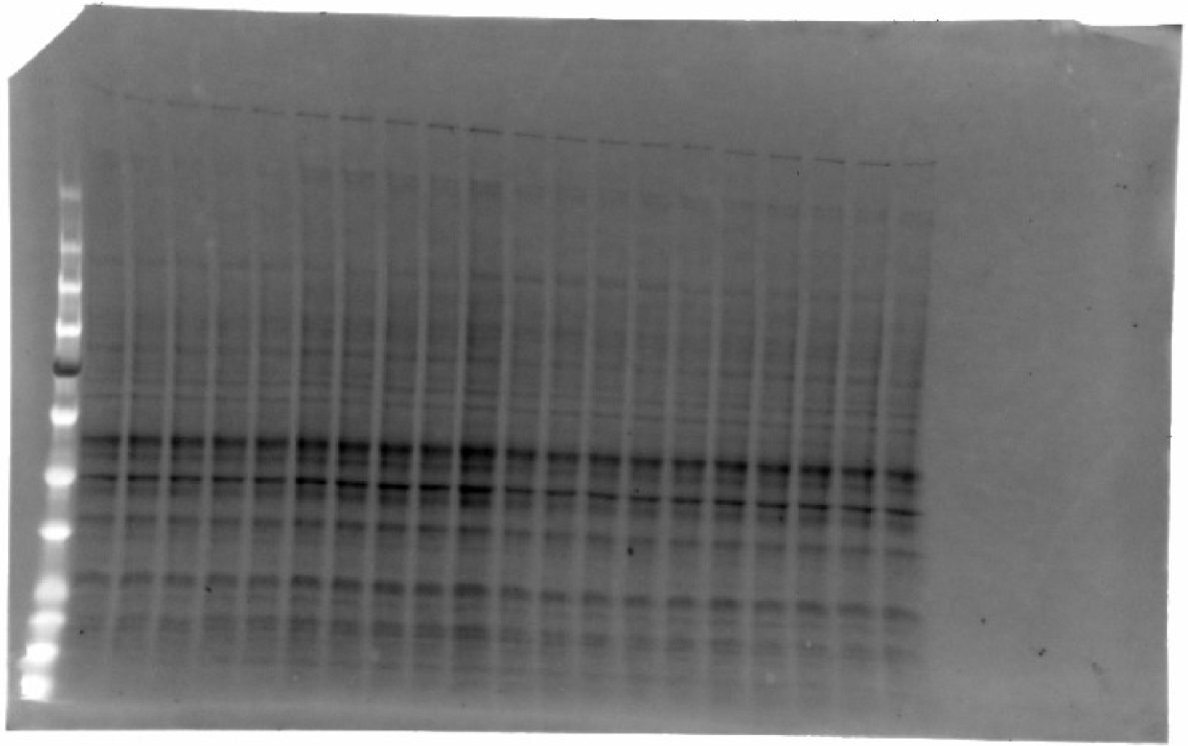

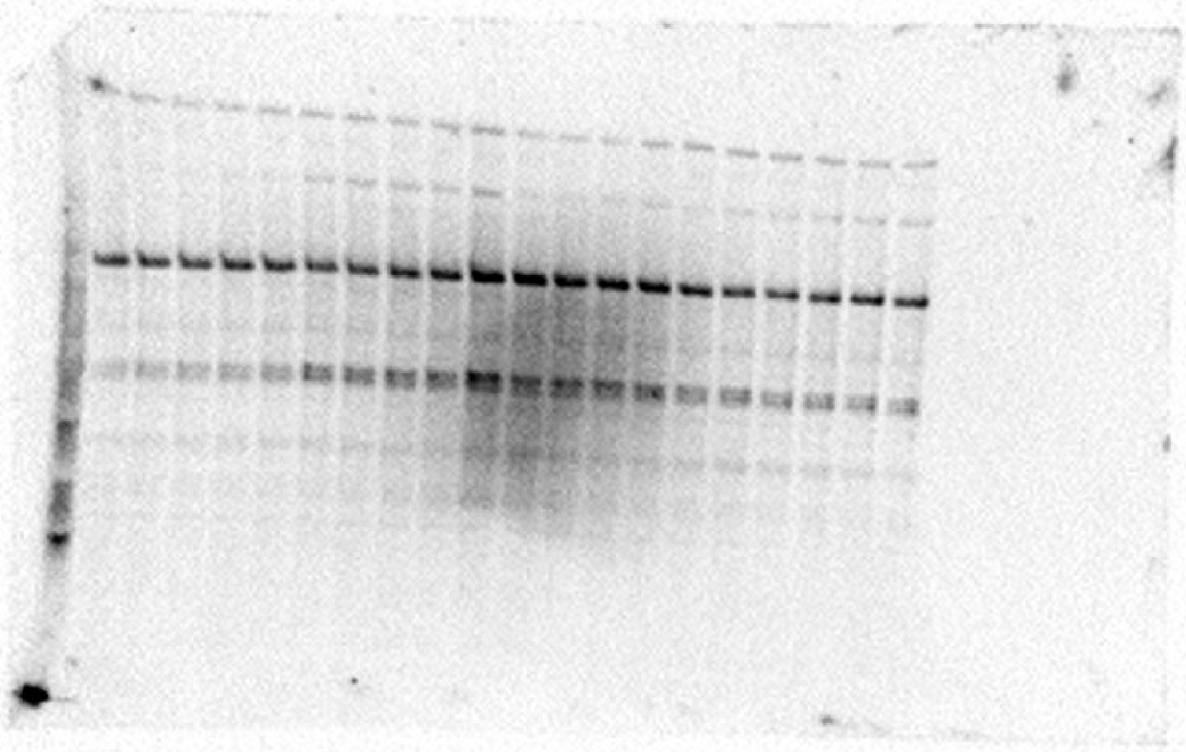

BCL2L13 MALE -

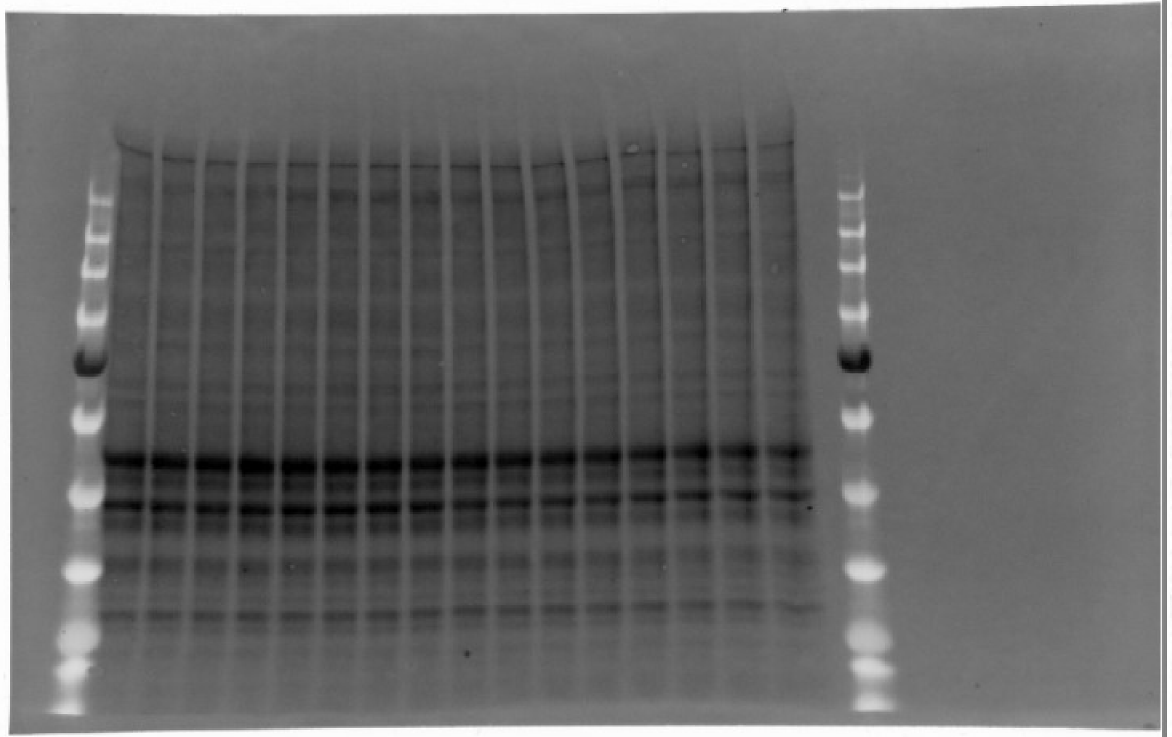

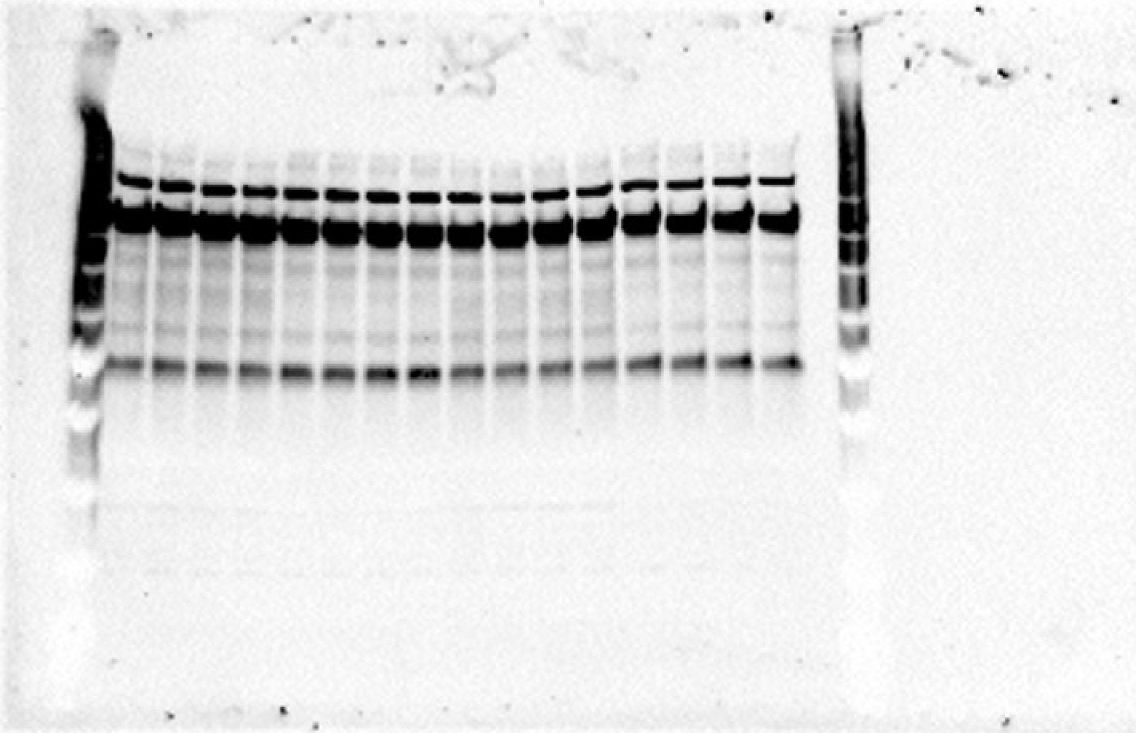

BNIP3 FEMALE-

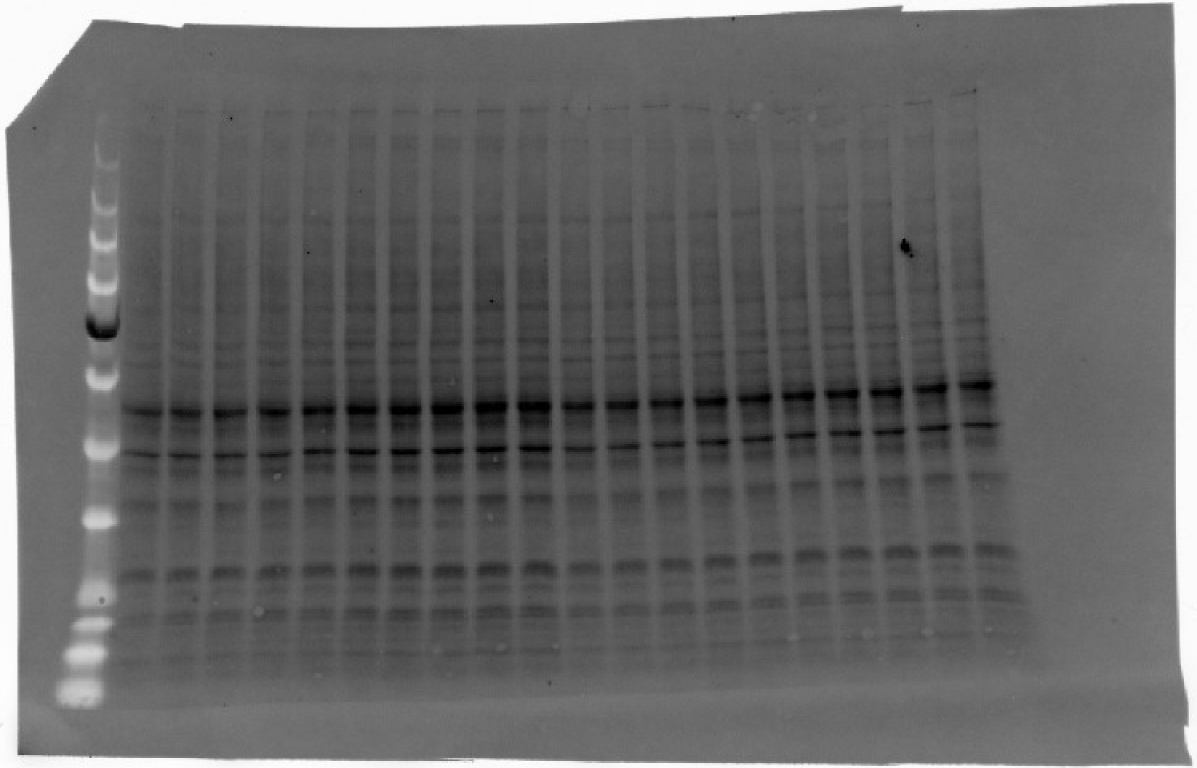

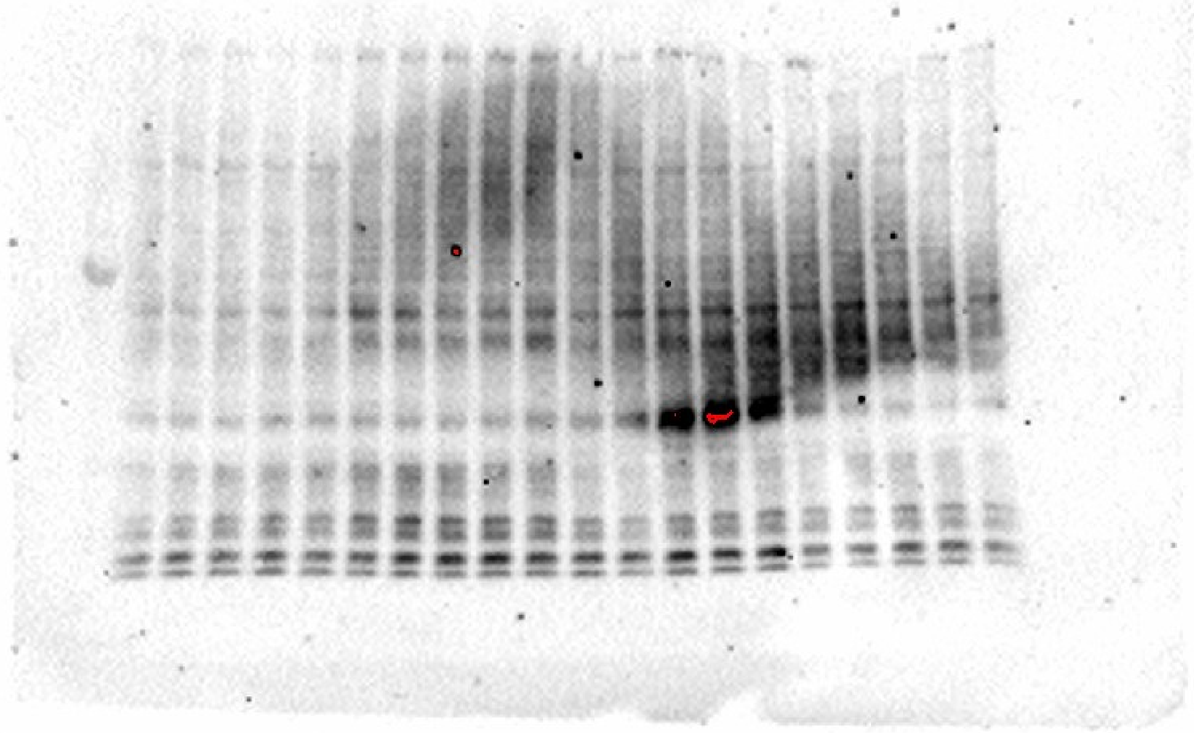

BNIP3 MALE-

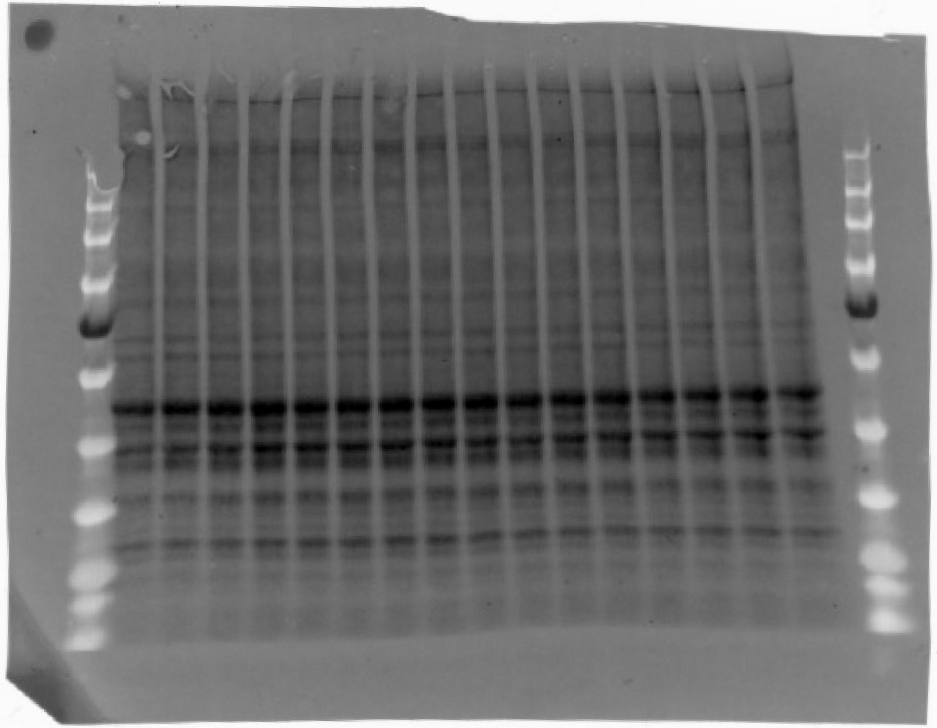

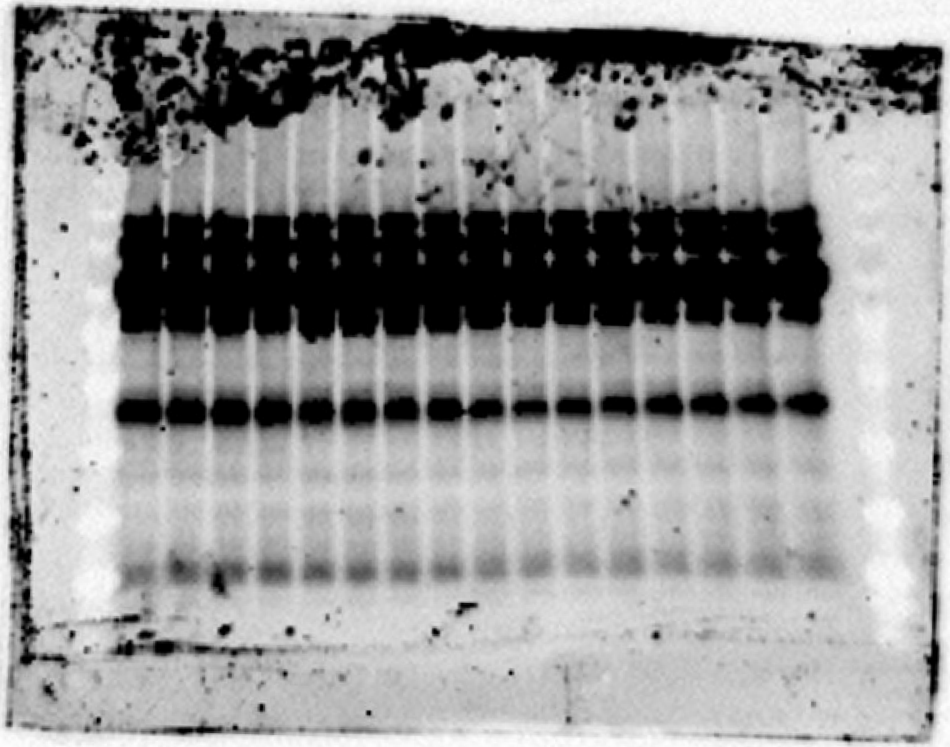

BNIP3L FEMALE-

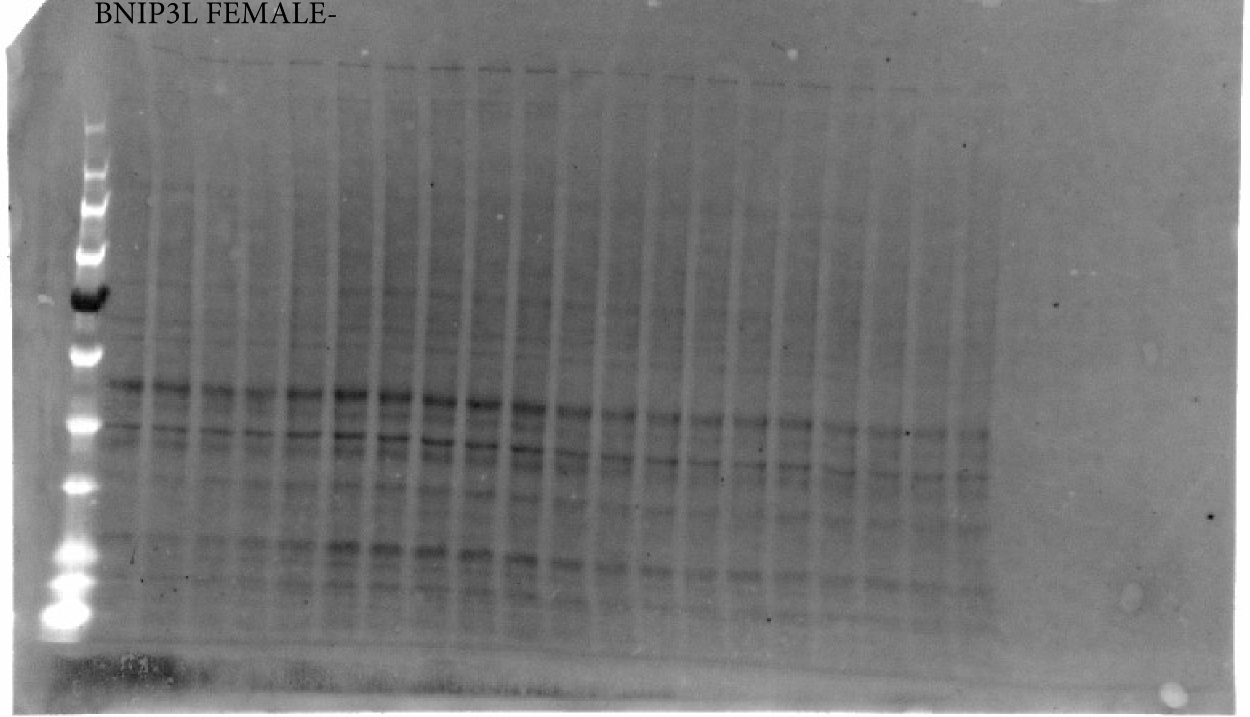

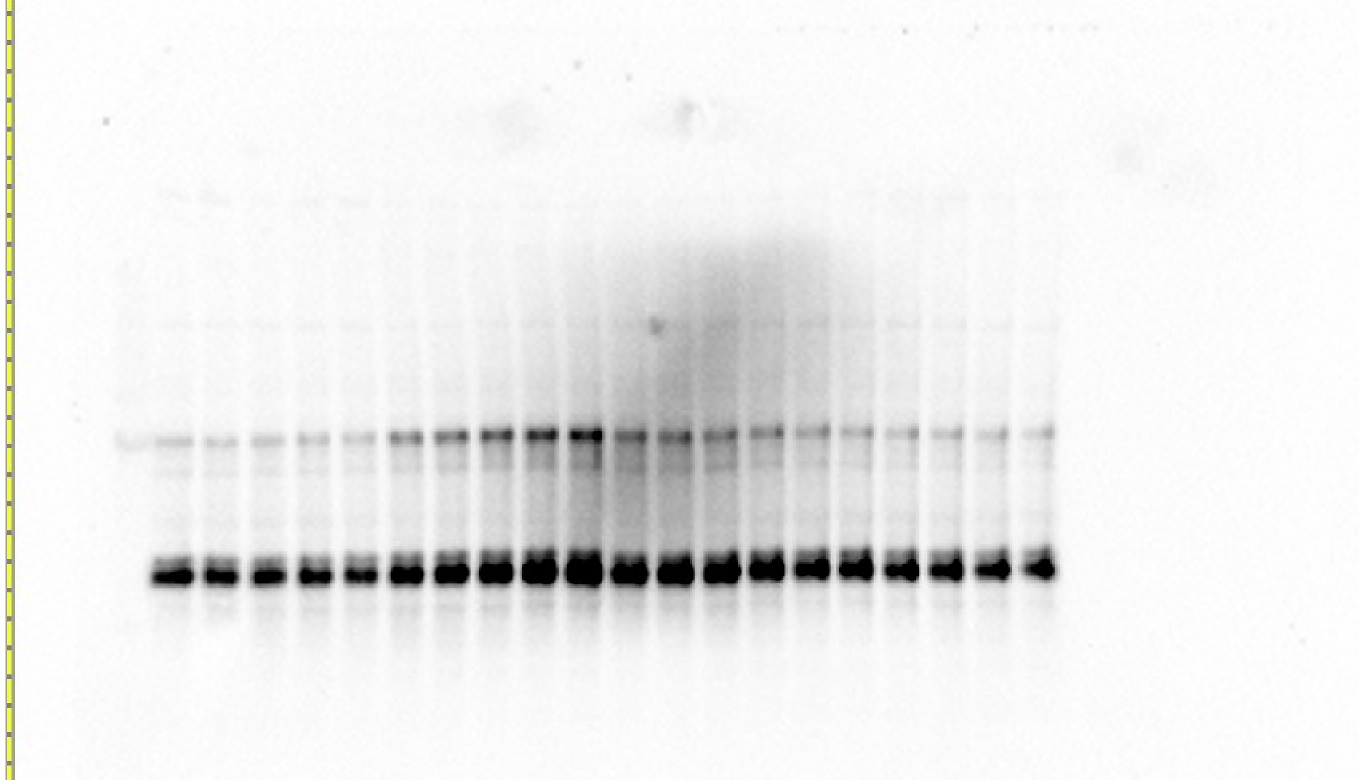

BNIP3L MALE-

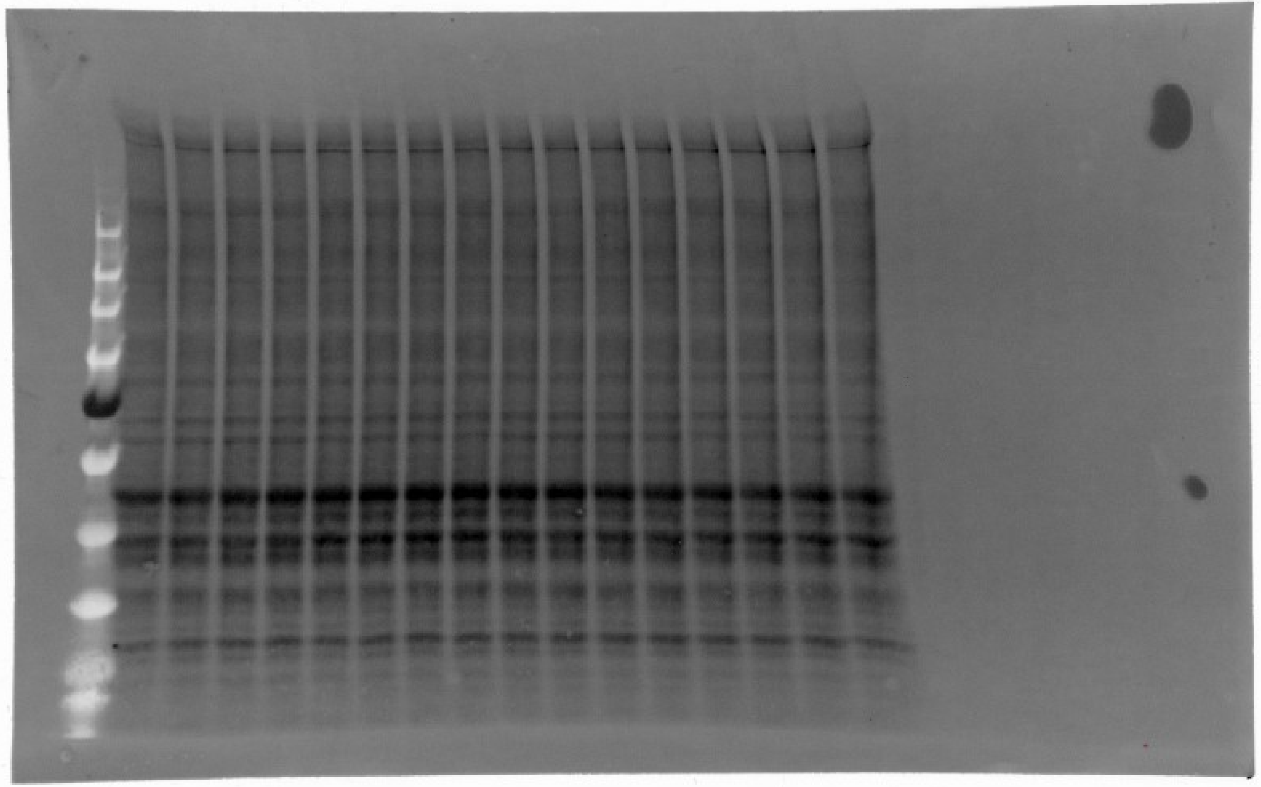

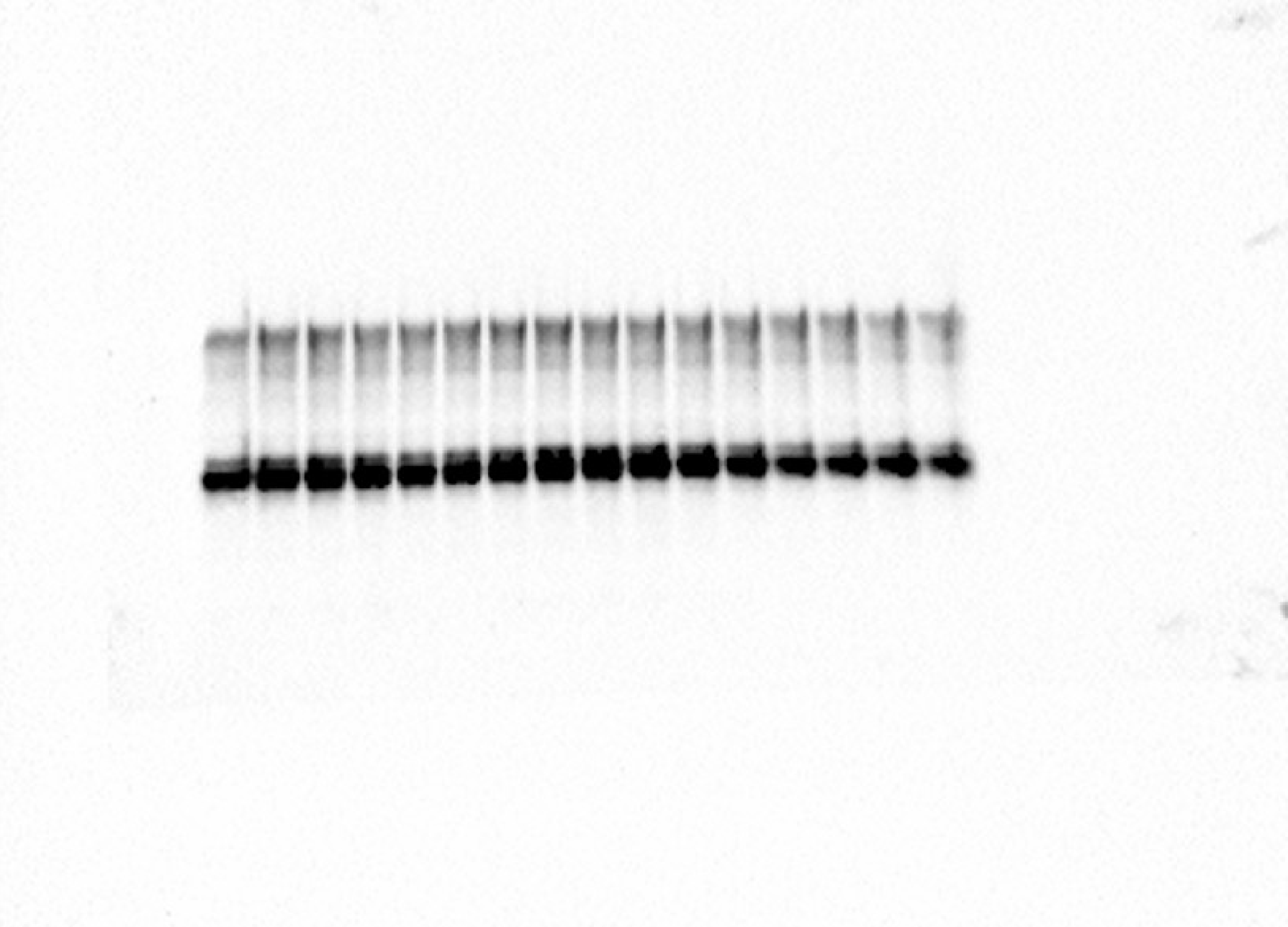

LC3 FEMALE-

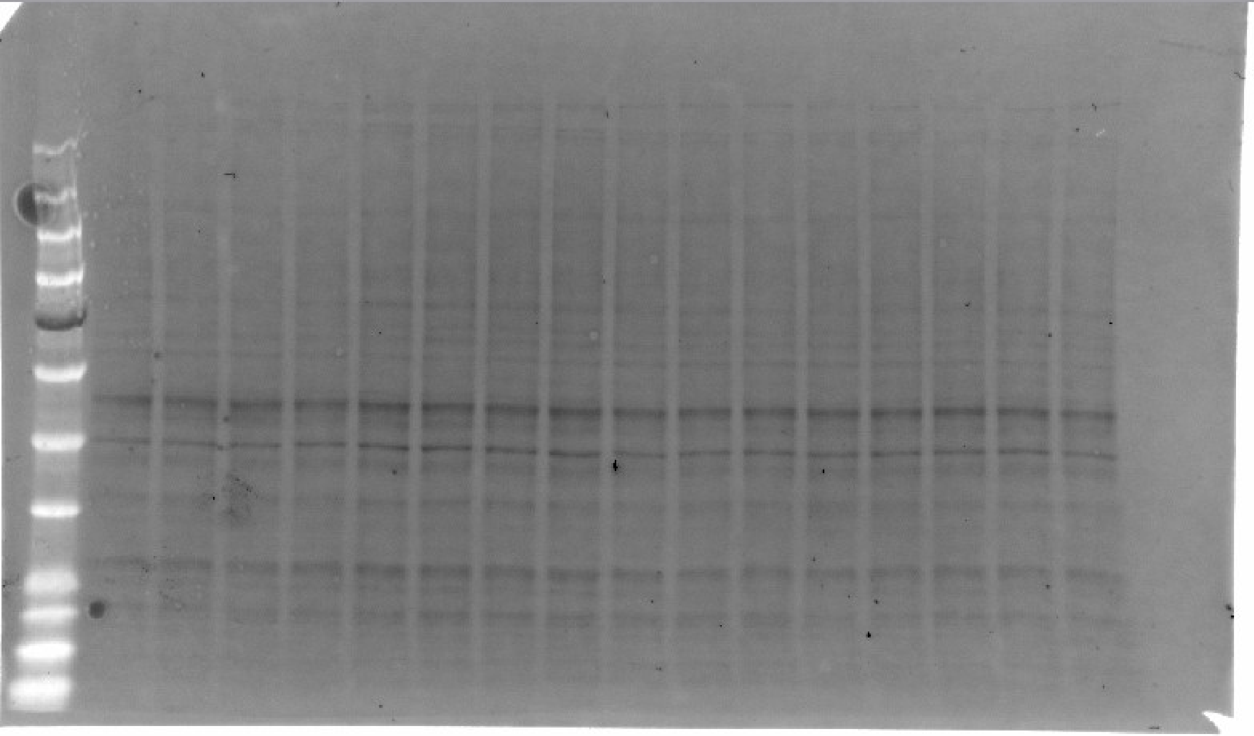

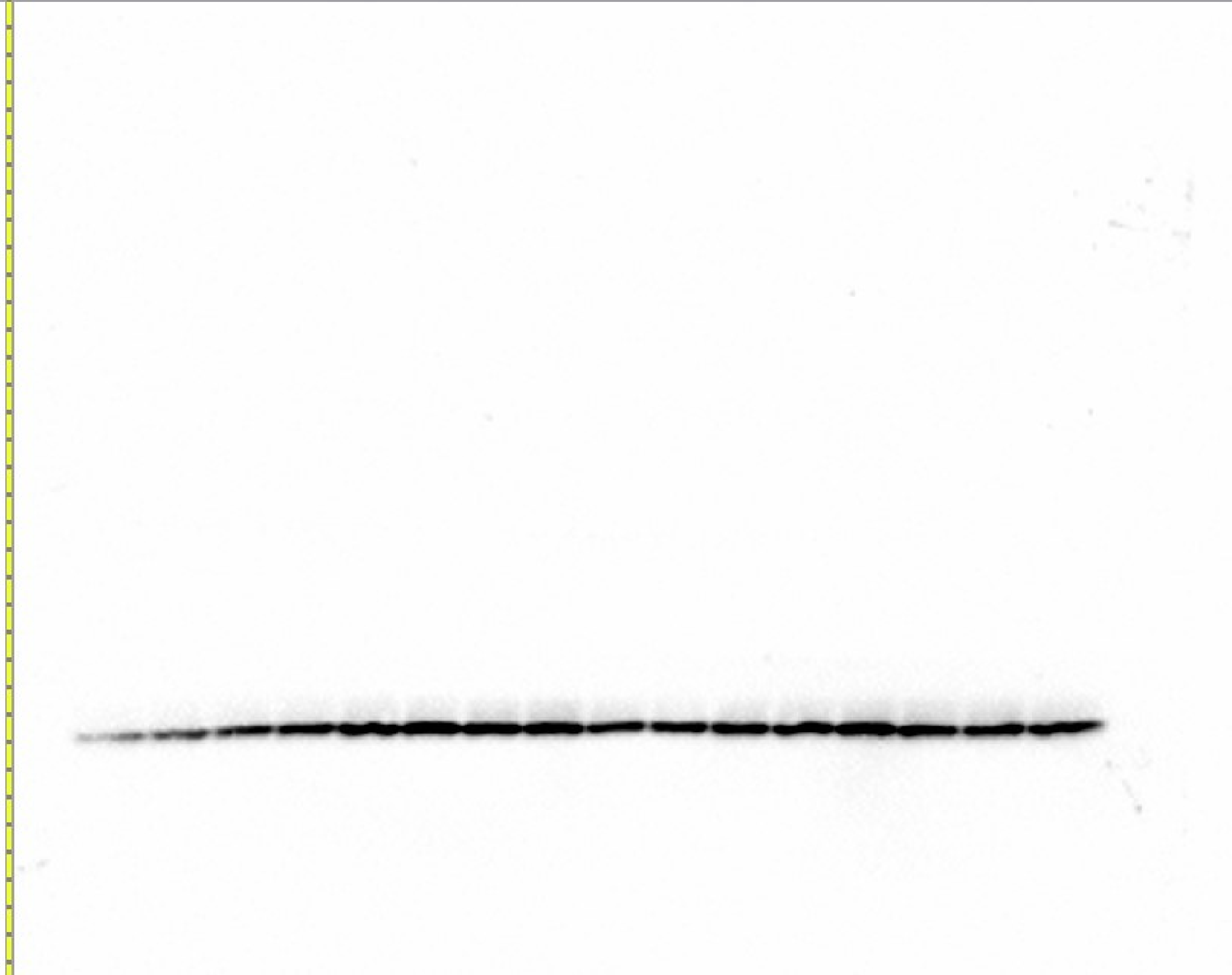

LC3 MALE-

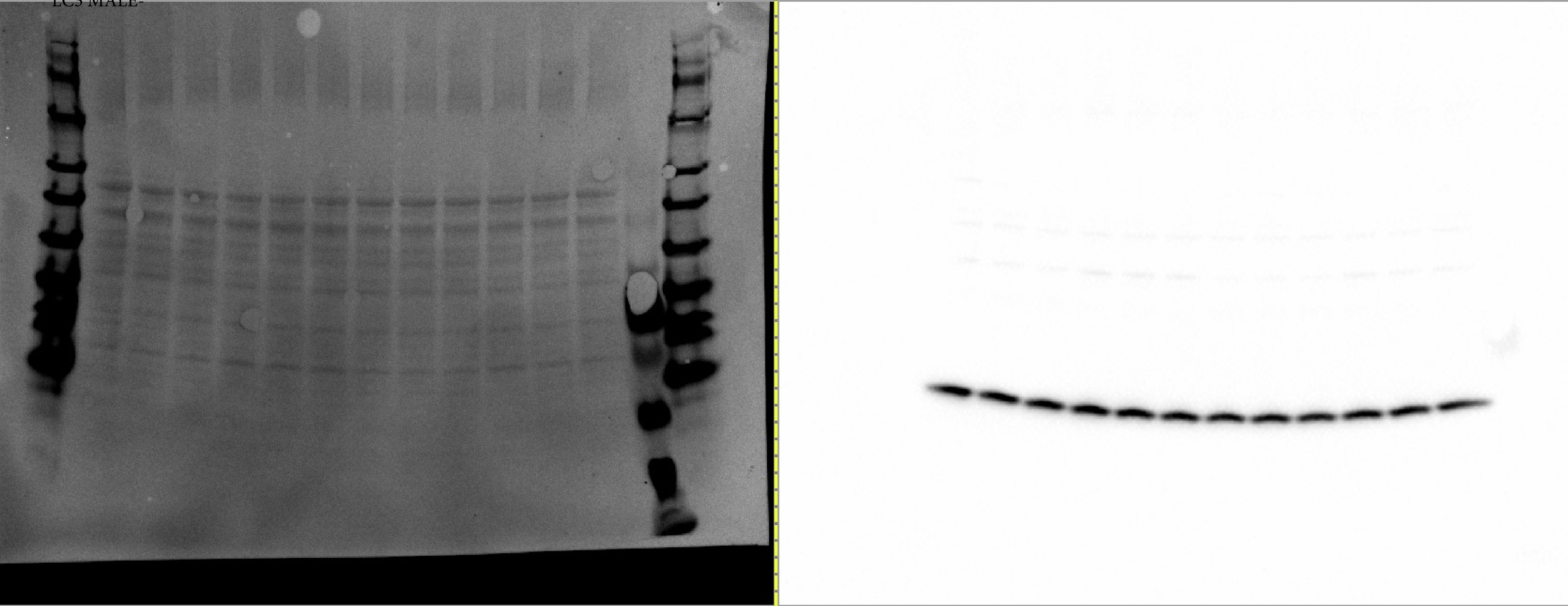

P62 FEMALE-

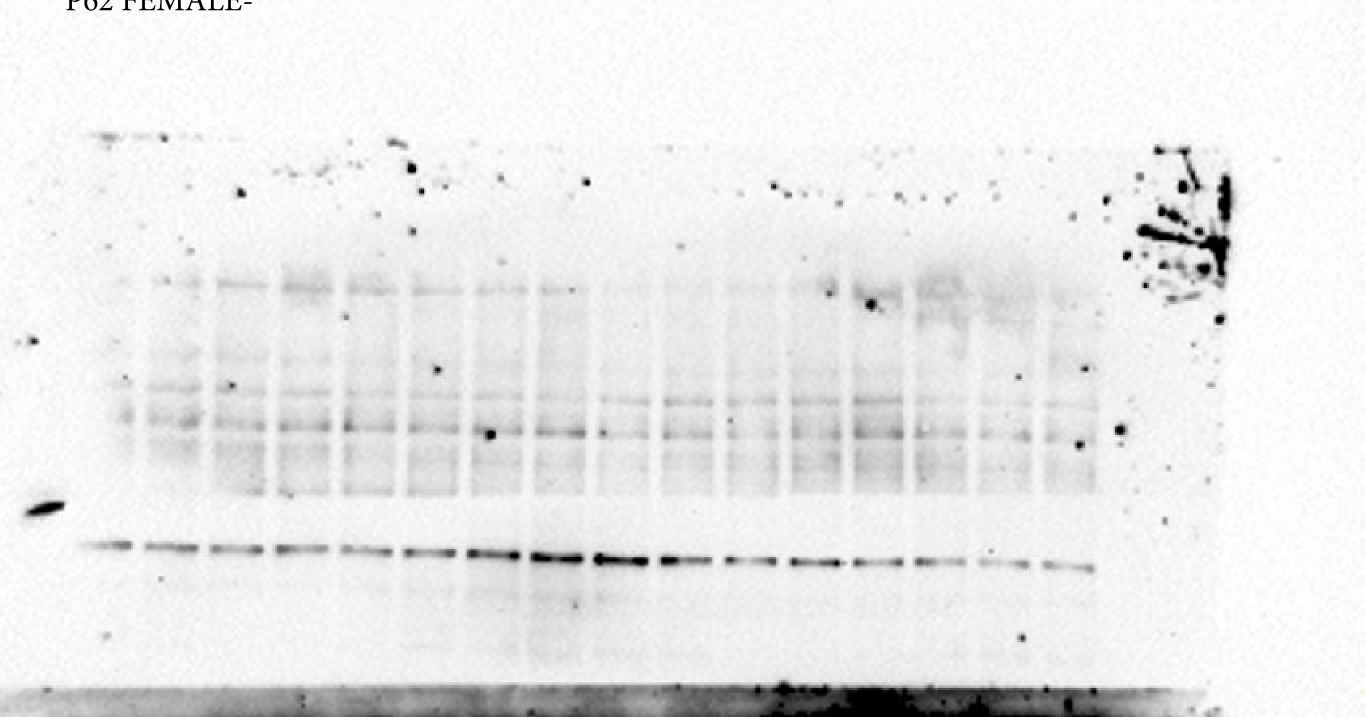

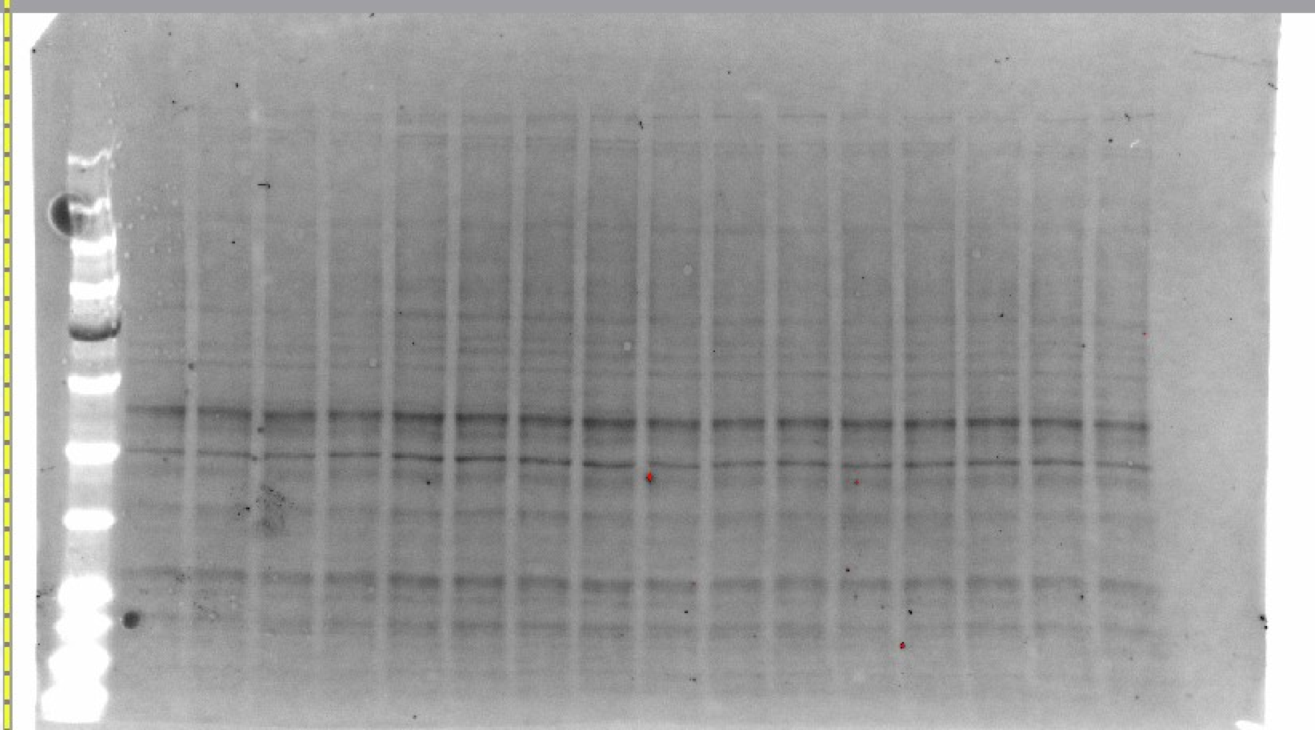

P62 MALE TOTAL PROTEIN

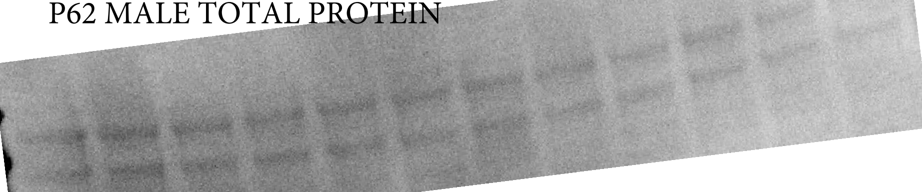

p62 MALE-

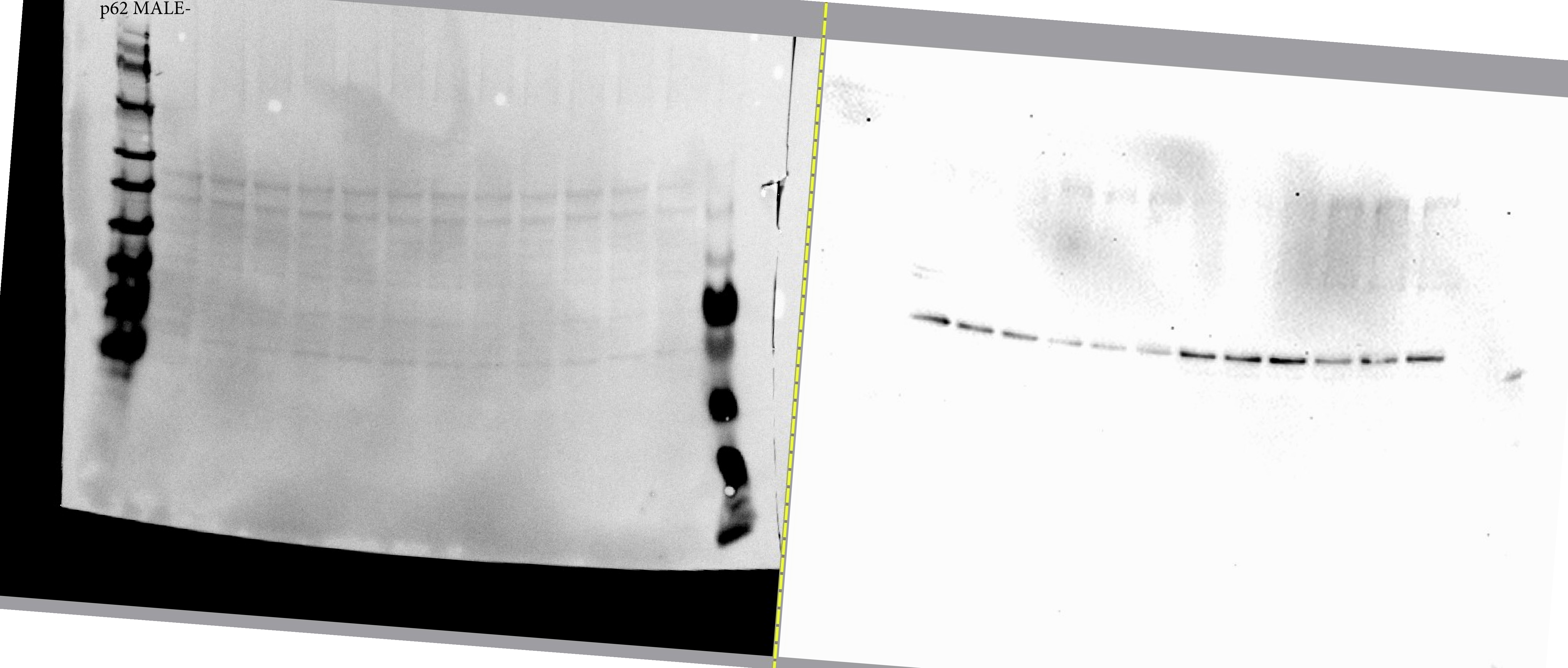

Supplement: Supplementary file 11 — Original Western Blot [file 41420_2025_2490_MOESM11_ESM.pdf]
